# Retained sponge after abdominal surgery: experience from a third world country

**Published:** 2009-07-05

**Authors:** Alain Chichom Mefire, Robert Tchounzou, Marc Leroy Guifo, Marcus Fokou, Jean Jacques Pagbe, Arthur Essomba, Eimo Elisée Malonga

**Affiliations:** 1 Faculty of health sciences, University of Buéa and regional hospital, Limbé, Cameroon; 2 General surgery unit, university teaching hospital, Yaoundé, Cameroon; 3 General and cardiovascular surgery unit, general and reference hospital, Yaoundé, Cameroon; 4 General surgery unit, central hospital, Yaoundé, Cameroon

**Keywords:** gossypiboma, abdomina

## Abstract

**Background:**

Retained abdominal sponge after surgery is a quite rare condition which can have heavy medico-legal consequences; its frequency is generally underestimated. Few reports of these conditions are available in African environment with specific technical and medico-legal background. We present our local experience of retained sponges after abdominal surgery and review current literature.

**Method:**

A retrospective analysis of the medical files of 14 consecutive patients with a retained surgical sponge after abdominal and urological surgery.

**Results:**

The incidence was 1every 677 abdominal operations; no metallic foreign body described, only sponges; the female sex predominated with 10/14 patients. 85.71% of retained sponge occurred after an emergency procedure and 64.28% were gynecological or obstetrical procedures. Most cases presented as intestinal obstruction, localized persistent pain or abdominal mass and pre-operative diagnosis could be done only in 28.57% of cases. A falsely correct sponge count was reported in 71.42% of cases 92.85% of patients were re-operated and the morbidity was low; no death was reported. None of our cases ended in a medico-legal claim despite proper counseling.

**Conclusion:**

The incidence of retained sponge might be significantly higher in an environment with reduced medico-legal threat; most cases of retained sponges are still related to human errors; the incidence will probably be reduced by a greater awareness about the condition.

## Background

Retained post-operative sponge, often referred to as “Gossipiboma”, is a quite rare condition. They are reported to occur in 1/1000 to 1/5000 operations [1, 2] . Their occurrence usually implies heavy medico legal problems as they may be presumably considered to be related to sub-standard surgical attention or negligence from the operative team [3]. It is suggested that this condition is grossly underestimated [4, 5, 6, 7, 8]; this could be related to the reluctance to report the incident because of its medico legal implications. The occurrence of Gossipiboma has been associated to some variables such as the emergency of the initial procedure, unexpected change in surgical procedure and patient’s body mass index [9].

The clinical and radiological presentation of Gossipiboma is often confusing, causing delay in diagnosis and proper management [8, 10, 11, 12]; this could end up in serious morbidity and mortality [4, 8].The diagnosis and management of this condition could be particularly challenging in an environment where the technical background is limited, especially concerning the possibilities of pre-operative diagnosis using imaging tools. We wish to share the experience from a third world country where the standards of practice and medico-legal implications could be very different.

## Methods

We retrospectively analyzed the files of 14 patients for whom a retained foreign body was discovered after a prior operative procedure during the period between January 1^st^ 1998 and December 31st 2004 in the 3 major university hospitals of the capital city of Yaoundé, Cameroon; these where central hospital, general reference hospital and university teaching hospital.; they all underwent an initial procedure including opening of the peritoneal cavity; one patient with a prior urological procedure without opening of the peritoneal cavity was also discovered.

The incidence of retained foreign body was estimated based on the total number of procedures with opening of the peritoneal cavity and urological procedures without opening of the cavity during the study period. For each of the 14 patients, we analyzed data regarding age and sex, nature and indication of previous operation, the number of previous operations, interval between probable causative operation and the discovery of the foreign body, clinical presentation, results of imaging procedures performed, tentative diagnosis, nature of foreign body discovered, procedure for removal and outcome; we also reviewed some of the known risk factors for retained foreign body as described in our study on available data. These data are discussed in the light of current literature and specificities of the environment of study.

## Results

The 14 cases of foreign bodies were part of a total of 9473 procedures fulfilling the inclusion criteria; they were described in 14 different patients, giving a rate of 1 foreign body every 677 operations; all foreign bodies discovered were a sponge: 12 ordinary sponges and 2 laparotomy pads; no metallic foreign body or other operative instruments were discovered. The ages ranged from 12 to 72 years with a mean age of 38.8 ± 13.66 years; there were 4 males and 10 females, giving a sex-ratio of 0.4/1. As shown on Table 1, the analysis of probable causative operation indicates that most cases (64.28%) were secondary to an emergency gynecological or obstetrical procedure; as a matter of fact, 12 procedures out of 14 (85.71%) were emergency procedures.

**Table 1:** Distribution of our patients according to type of causative operations

**Type of causative operation**	**Number**	**Percentage**
Emergency Caesarean section	5	35.70
Laparotomy for ruptured ectopic pregnancy	4	28.55
Laparotomy for peritonitis of appendicular origin	1	7.15
Laparotomy for duodenal ulcer perforation	1	7.15
Emergency splenectomy	1	7.15
Transvesical prostate adenomectomy	1	7.15
Non complicated inguinal hernia repair	1	7.15
**Total**	**14**	**100**

The interval between probable causative operation and discovery of foreign body ranged between 2 and 206 weeks, with a mean of 6.12 ± 89.43 weeks. Table 2 displays the clinical presentation of each case; it indicates that the most frequent presentations were intestinal obstruction (42.85%), localized persistent abdominal pain (21.42%) and abdominal mass (14.28%). All cases of intestinal obstruction were diagnosed as adhesive obstruction on clinical basis. Abdominal echography was performed in 11 cases and suspected a foreign body in 2 cases; standard X ray of the abdomen performed in all patients was unproductive. A CT scan was performed in one patient and suspected a tumor of the right colon; this concerned one case of prior laparotomy for peritonitis of appendicular origin.

**Table 2:** Distribution of our 14 cases according to probable causative operation, clinical background and tentative diagnosis

**patient**	**Causative procedure**	**Clinical presentation**	**Tentative diagnosis**
**1**	Emergency C/S^1^	intestinal obstruction	Adhesive obstruction
**2**	Emergency C/S^1^	intestinal obstruction	Adhesive obstruction
**3**	Emergency C/S^1^	Localised persistent abdominal pain	Retained sponge
**4**	Emergency C/S^1^	Abdominal mass	Retained sponge
**5**	Emergency C/S^1^	intestinal obstruction	Adhesive obstruction
**6**	Laparotomy for REP^2^	undescribed	undescribed
**7**	Laparotomy for REP^2^	Localised persistent abdominal pain	
**8**	Laparotomy for REP^2^	intestinal obstruction	Adhesive obstruction
**9**	Laparotomy for REP^2^	intestinal obstruction	Adhesive obstruction
**10**	Laparotomy for DUP^3^	Abdominal mass	Tumour
**11**	TVPA^4^	Incomplete urinary retention	Urethral stenosis
**12**	Laparotomy for GPAO^5^	intestinal obstruction	Adhesive obstruction
**13**	Hernia repair	Discharging sinus	Retained sponge
**14**	Emergency splenectomy^*^	Localised persistent abdominal pain	Retained sponge

1 C/S: caeserian section

2 REP: ruptured ectopic pregnancy (all where located in the tubes)

3 DUP: Duodenal ulcer perforation

4 TVPA: transvesical prostate adenomectomy

5 GPAO: generalized peritonitis of appendicular origin

* the splenectomy was performed for a traumatic splenic rupture

A retained sponge was diagnosed pre operatively in 4 cases (28.57%). The diagnosis was based on association of clinical and echographical arguments; in one of these cases, an incorrect sponge count was reported during the causative operation. All patients diagnosed preoperatively were readmitted less than 6 weeks after probable causative operation. 13 patients (92.85%) required an operation; one patient was not operated; this was a 69 years old patient who previously underwent a trans-vesical prostate adenomectomy and who spontaneously expelled the sponge by urination during radiological investigations. No post operative death was reported. One patient developed a post operative external fistula from small bowel which was managed conservatively.

Some of the risk factors described in current medical literature were identified and described as shown in Table 3; the most frequent risk factor was the causative operation performed as an emergency; a falsely correct sponge count was also reported in 71.42% of cases. It must be outlined that 3 causative operations (21.42%) were performed by a resident under supervision. 2 repeated offenders were identified in our study, namely one senior gynecologist who accounted for 4 retained sponges in 4 years (one per year!), and one senior surgeon who was found to be responsible of two retained sponges in one year, including one laparotomy pad.

**Table 3:** Review of some of the known risk factors as described in our study

**Risk factor**	**Number of cases**	**Percentage**
sponge count not done	1	7.15
incorrect sponge count reported	1	7.15
correct sponge count reported	10	71.42
confusing bleeding during operation	1	7.15
operation performed as an emergency	12	85.71
resident as main operator (under supervision)	3	21.42
Patient’s body mass index above 40	1	7.15
Operation performed late in the night (after midnight)	7	50

## Discussion

The rate of retained surgical sponge after abdominal surgery is estimated to range between 1/1000 to 1/6000 operations [2, 4, 5, 6, 9]; the rate described in our study seems to be higher than what is generally reported. It is clear in most recent publications that the rate described is grossly underestimated [4, 6, 7, 8, 13]; reasons for this are related to the possible medico-legal implications, the fear of litigation which could end up in heavy expenses for compensations [1] and adverse publicity for institutions and surgeons; in fact, it is clear that the responsibility of the surgeon and members of the team in the theater could be called in case of litigation [3].

In our environment, there are at least 2 possible reasons why the rate in our report could be so high: it is a third world country were the standards of medical care and technical background are generally below what is considered normal in developed countries; recommended radiopaque sponges which are known to ease the identification of sponge are not available [14, 15] and equipment for intra-operative X-ray search of forgotten sponge is not available in most places. However, it must be outlined that more than 75% of surgeons in the world practice under such conditions! On the other hand, concerning the medico-legal aspect, the local laws define responsibilities and compensation mechanism following what is described as medical negligence; but, despite the fact that all our patients and/or their relatives were informed of the findings, none of our cases resulted in malpractice claim! Many recent studies are based on statistics of malpractice claims which are likely to be underestimated because they will include operations without cavity opening which have very little chance of resulting in a forgotten instrument [9]. This is an indication that the rate for abdominal procedures which involve the opening the peritoneum (one of our inclusion criteria) is expected to be significantly higher.

Sponges represent 50 to 65% of retained material after surgery [2, 4, 9]; materials such as clamps and retractors have been described [4, 9]. In our study no metallic instrument or other foreign material was recorded.

The female preponderance in our study has been described by other authors [6, 9], and is probably the reflection of the preponderance of gynecological procedures as causative operations; but female sex has not been identified as a risk factor [9]. In the vast majority of our patients, the probable causative operation was performed as an emergency; emergency procedures have been clearly identified as a major factor for retained material after surgery [2, 4, 6, 9]. According to Bani-Hani et al., emergency procedures increase the risk of leaving o foreign body behind by nine [6]. It has also been suggested that the rate of retained sponge increase during war, because the work is done under pressure, strenuous conditions with a great inflow of wounded people [16].

The clinical presentation and working diagnosis in our study is quite similar to data of current literature [7, 8, 10, 17, 18]; the delay between causative operation and manifestation of the retained sponge could be as high as 206 weeks (almost 4 years); this delay is known to be very variable [7, 16, 19, 20]; Klaric et al. diagnosed 11 textilomas in 10 patients within 3 months following the causative abdominal surgery [16]; durations as long as 40 years have been described [20]. Occlusive, septic and pseudo-tumoral presentations displayed by our study are the most frequently described situations at various ranges [4, 5, 7, 19]; according to Schönleben et al, metallic instruments will cause more acute symptoms in an earlier time than sponges [4]. Most retained sponges will be symptomatic when discovered; Yildirim et al. described 1 asymptomatic case in 14 cases (7%) [10]. The preoperative diagnosis of retained sponge is not obvious; less than 30% in our study, it usually ranges between 35 and 100% [6, 8, 10, 18]. The clinical assessment is clearly not specific, but characteristic ultrasound and computed tomography images have been described [11].

The use of imaging tools is likely to improve the rate of preoperative diagnosis; ultrasound could be of serious help; according to Stanciu et al., echography is not very specific, but could be highly suggestive when the clinical background elicits suspicion[12]; a combination of clinical and echographical arguments diagnosed a foreign body successfully in more than 65% of cases in the study of Talciyldiz et al. [18]; in the contrary, the judicious combination of clinical examination, abdominal X-ray, echography, computed tomography, magnetic resonance imaging and upper gastrointestinal endoscopy successfully diagnosed retained sponge in only 36% of cases in the study of Yildirim et al. [10]. The extended use of radiopaque sponges does not guaranty and improved preoperative diagnosis rate; Bani-Hani et al. diagnosed the forgotten sponge on plain X-ray only in only 18% of cases [6]. The rate of preoperative diagnosis could in fact be better improved by an increased awareness about the possibility of a retained sponge; this diagnosis must systematically be included in the differential diagnosis of every patient who develops an occlusive syndrome, an abdominal mass, a fistula or another septic complication after abdominal surgery. Some authors have described cases where laparoscopy was used as a tool for diagnosis and management [21]. Another identified risk factor is a falsely correct sponge count; this was reported in more than 70% of our cases; Bani-hani et al. described it in 72% of their cases [6]; the findings of Kaiser and al where almost identical [1]; however, Gawande et al. did not describe a correct sponge count as a significant risk factor in a case control study [9].

One case of incorrect sponge count was identified in our study; the use of radiopaque sponges, though not a guarantee [6], could help solve the problem; but if the sponge is not found after exploration of the abdomen by at least 2 independent senior surgeons as we do in our practice, it can be an embarrassing situation. Performing an abdominal surgery without a reported sponge count must be considered unacceptable nowadays. Operations after hours and involvement of residents have been analyzed and are generally not considered as significant risk factors [9]. In particular, emergency operations are known to be frequently performed by residents and junior surgeons to a large extend [6]. We do not wish to display in this report our local measures against repeated offenders, but it seems they are not sufficient to discourage some of them as one operator alone recorded 4 cases in 4 years; there is clearly indication that tougher measures must be considered in our environment against repeated offenders. In almost all cases, forgotten sponges will end up in a re-operation when the causative operation requires opening of a cavity [2, 6, 8, 10, 13, 17, 18]; non operative management is usually the result of spontaneous self-extrusion of the retained sponge which is frequently described [6, 19, 22]; our spectacular and painful urination of a sponge is at our knowledge un-described so far. The morbidity after surgical management of a retained sponge could unfortunately be high, ranging from 10 to 50% and consisting mainly of infection and abdominal wall complications [8, 10]; this could even end up into death in rates as high as 35% [4, 8]; however, low complication rates and no mortality as described in our study have also been reported [2, 18].

## Conclusion

No rate of retained foreign body can be considered “acceptable” whatever the environment and conditions of work; their consequences in terms of morbidity and mortality can still be too heavy and costly. Their management will still rely for a long time on prevention because in almost all cases, it could be related to human errors; this type of errors will probably never be completely abolished, but the incidence of retained surgical sponge can be reduced to a “minimum” by strict adherence to regulations, especially systematic and rigorous sponge count; this is particularly important during emergency procedures. Some surgeons have reduced the risk by advocating systematically against the use of sponges in an open cavity. Their early diagnosis will probably be improved if it is based on a greater awareness of the possibility of retained foreign body in every suggestive post-laparotomy complication, rather than on imaging techniques, especially in environment with limited technical background.

## Competing interest

The authors declared they have no conflicts of interest.

## Authors’ contribution

ACM insure the overall coordination of the study. All the authors have read and approved the final version of the manuscript.
